# Challenges in Glaucoma Management in High Myopia After Refractive Surgery: A Case Report

**DOI:** 10.7759/cureus.100859

**Published:** 2026-01-05

**Authors:** Rodrigo Brazuna, Dillan Cunha Amaral, Giovanni Nicolla Umberto Italiano Colombini, Marcella Q Salomão, Renato Ambrósio

**Affiliations:** 1 Department of Ophthalmology, Federal University of the State of Rio de Janeiro, Rio de Janeiro, BRA; 2 Faculty of Medicine, Federal University of Rio de Janeiro, Rio de Janeiro, BRA; 3 Department of Ophthalmology, Federal University of São Paulo, São Paulo, BRA

**Keywords:** biomechanically corrected iop, corvis, corvis st, glaucoma, high myopia, intraocular pressure, keratoconus, radial keratotomy

## Abstract

Managing glaucoma in highly myopic patients who have had prior refractive surgery is particularly complex. The altered corneal biomechanics in these eyes interfere with standard intraocular pressure (IOP) assessment, making conventional measurement less reliable. This report presents a 52-year-old male athlete with high myopia, who underwent radial keratotomy 25 years ago and evolved to iatrogenic ectasia on the OD (right eye), treated with intra-stromal ring implantation. Several years later, he underwent bilateral cataract surgery, followed by laser retinopexy and vitrectomy for sequential retinal detachment. Goldmann applanation tonometry (GAT) showed a stable mean of 15 mmHg in the OS (left eye), whereas readings in the OD varied widely, from 18 to 50 mmHg, depending on the applanation site, likely due to corneal scarring and the presence of the Ferrara ring. IOP measured with Corvis ST remained elevated in both eyes (29 mmHg OD and 28 mmHg OS). OCT revealed retinal nerve fiber layer (RNFL) thinning, and a visual field defect was also documented with the central 10-2 Humprey Field Analyzer with the 10-2 protocol and SITA-Standard strategy. Despite long-term treatment, clinical control was insufficient, as progression was documented on 10-2 visual fields, symptom deterioration, and biomechanical evidence suggesting sustained IOP-related damage, even where numerical values were inconsistent. We therefore performed two sessions of micropulse transscleral cyclophotocoagulation. This intervention led to a moderate reduction in IOP and appeared to slow the progression of both structural and functional changes. This report highlights the limitations of GAT in eyes with a history of refractive surgery, particularly radial keratotomy, as well as in ectatic disorders and patients with Ferrara’s ring implantation. It also emphasizes the value of corneal-independent methods for tonometry combined with functional monitoring. In situations where surgical intervention is either not the most appropriate indication or cannot be feasibly performed, micropulse laser offers a viable and effective alternative. Individualized glaucoma management remains essential in post-refractive, myopic patients with structurally abnormal eyes.

## Introduction

Glaucoma management in patients with high myopia who have undergone corneal refractive surgery is notably complex [1]. High-degree myopia increases the risk of primary open-angle glaucoma, with epidemiologic studies showing a several-fold higher incidence of glaucoma in these eyes [2]. Accurate intraocular pressure (IOP) measurement is critical for glaucoma diagnosis and management, yet obtaining a reliable IOP in post-refractive surgery corneas is challenging [3,4]. Radial keratotomy (RK) and other myopic refractive procedures permanently alter corneal structure and biomechanics, often leading to underestimation of IOP by Goldmann applanation tonometry (GAT) [3-5]. Because GAT relies on assumptions regarding corneal thickness, rigidity, tangent curvature, hydration, and the cornea’s viscoelastic behavior, alterations in any of these parameters lead to systematic underestimation of IOP. Highlighting these biomechanical dependencies underscores why GAT remains vulnerable to error despite its widespread clinical use.

In eyes that have undergone laser-assisted in situ keratomileusis (LASIK), photorefractive keratectomy (PRK), or RK, the central cornea becomes thinner or biomechanically weaker, so the force required to applanate 3.06 mm is lower than assumed by GAT, yielding an erroneously reduced IOP reading [3]. Corneal ectatic conditions, such as keratoconus or iatrogenic ectasia, after laser vision correction (LVC) further exacerbate this inaccuracy [6]. Because RK creates deep radial incisions that weaken the cornea and Ferrara intrastromal ring segments locally increase stromal stiffness, these interventions produce a heterogeneous biomechanical profile that adds additional variability to applanation-based tonometry [3-5].

Studies in keratoconic eyes have shown that GAT readings are significantly lower than true IOP, whereas newer devices, less dependent on pachymetry and curvature, provide higher (more accurate) IOP values [3]. An interesting report found that post-LASIK myopic eyes showed approximately 5 mmHg drops in GAT-IOP while the true IOP, measured by dynamic contour tonometry, remained unchanged [3]. These findings indicate that corneal-dependent tonometry may be unreliable after refractive surgeries such as IntraLASIK and LASEK [3]. To address this problem, various “cornea-independent” tonometry methods have been developed and proposed [4]. Recently, the Corvis ST (Oculus GmbH, Wetzlar, Germany) tonometry system was introduced. The system provides a biomechanically corrected IOP (bIOP), which incorporates the cornea’s dynamic deformation response to provide a more reliable IOP [6,7]. This bIOP is designed to be minimally influenced by corneal thickness and by the cornea’s biomechanical properties [5,8,9]. Early clinical data suggest that bIOP measurements remain closer to true IOP in post-refractive eyes, whereas GAT significantly underestimates IOP [3,4].

Although intra-stromal corneal ring segments, such as Ferrara’s ring, are known to locally increase corneal stiffness near the site of implantation, this biomechanical modification does not appear to result in a clinically relevant overestimation of IOP when measured centrally using GAT [10].

High myopes often have large, tilted optic discs and thin scleral canals, which can hinder optic nerve evaluation [2]. Therefore, in high myopic eyes submitted to LVC procedures that alter IOP readings, computerized visual field testing is crucial [8].

Herein, we present a complex case of glaucoma in a highly myopic eye submitted to RK, who subsequently developed corneal ectasia. This case report shows the great difficulty in IOP assessment and the tailored interventions employed, including the use of advanced tonometry and micropulse cyclophotocoagulation, to achieve glaucoma control in the context of an altered cornea. The coexistence of high myopia, prior RK, progressive ectasia, a Ferrara ring segment, and glaucoma is exceptionally uncommon, creating a unique biomechanical environment that significantly complicates both diagnosis and management.

## Case presentation

A 52-year-old male academic and active martial-arts practitioner from Brazil. He was referred for evaluation due to a complex glaucoma management in the context of high myopia and prior refractive and retinal surgeries. A chronological summary of key interventions and IOP readings is provided in Table 1.

**Table 1:** Clinical timeline and ocular parameters. GAT, Goldmann applanation tonometry; IOP, intraocular pressure; mmHg, millimetres of mercury; OD, oculus dexter (right eye); OS, oculus sinister (left eye)

Intervention	OD IOP (GAT) (mmHg)	OD bIOP Corvis (mmHg)	OS IOP (GAT) (mmHg)	OS bIOP Corvis (mmHg)
Month 0: Initial evaluation	18-36	32	16	30
Month 1: Follow-up	14-34	29.1	12	28
Month 2: Vitrectomy + gas (OD)	25-38	28	14	12
Month 3: Post-op vitrectomy + gas (OD)	32-50	25	14	12
Month 4: First micropulse G6 laser	10-28	24	15	12
Month 5: Post-laser follow-up	10-28	24	15	11.5
Month 13: Follow-up	24-48	38	16	12.5
Month 14: Post-second G6 laser session	20	16.1	18	15

The patient had undergone RK approximately 25 years earlier in both eyes for high myopic correction. He later developed corneal ectasia in the right eye (OD), which was managed with implantation of a Ferrara intracorneal ring segment. He reported bilateral phacoemulsification with intraocular lens implantation several years before presentation. He had also undergone multiple laser retinopexies for peripheral retinal breaks in both eyes. At the time of his initial evaluation, he was using Ganfort® (bimatoprost 0.03% + timolol maleate 0.5%) and Glaub MD® (brimonidine tartrate 0.2%) for glaucoma management.

**Figure 1:**
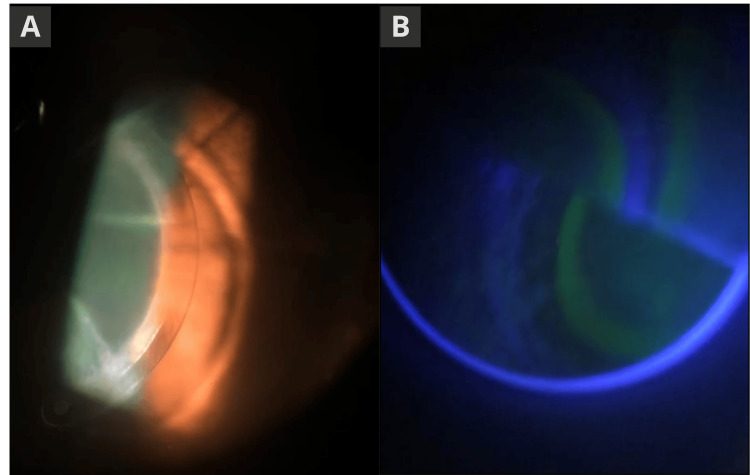
Corneal alterations and intraocular pressure measurement in the right eye. (a) Slit-lamp photograph of the right eye showing radial keratotomy scars and the Ferrara intrastromal corneal ring; (b) Goldmann applanation tonometry image of the OD demonstrating variability in applanation zones due to corneal irregularities.

At the first presentation, best-corrected visual acuity (BCVA) was 20/40 in OD (refraction +7.75DS/-4.50DC × 90, determined by subjective refraction for spectacle correction) and 20/30 in the OS (left eye), with no improvement after refraction. In OD, GAT showed highly variable IOP readings, ranging from 18 to 36 mmHg depending on the site of applanation, clearly influenced by previous RK incisions and the intracorneal ring. The OS measured 16 mmHg. Corneal topography and slit-lamp examination confirmed RK scars and pseudophakia in both eyes, with a Ferrara ring segment in OD, and additionally demonstrated asymmetric bow-tie distortion with incision-related flattening, paracentral steepening adjacent to the ring segment, and residual irregular astigmatism. Gonioscopy showed open angles and dense 360° trabecular meshwork pigmentation. Representative slit-lamp and GAT images of OD are shown in Figures 1a-1b.

**Figure 2:**
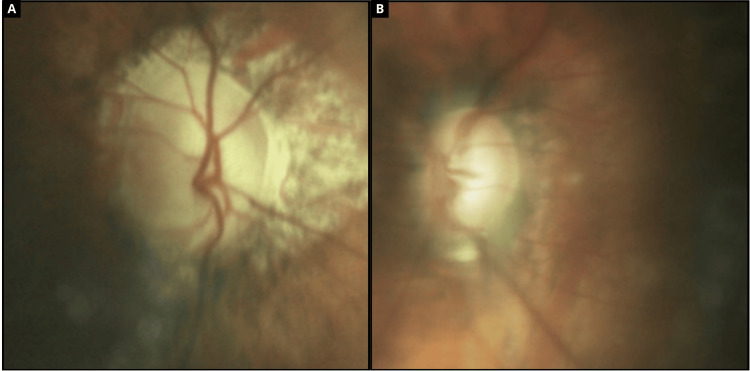
Color fundus photography. (a) OD with tilted optic disc, advanced cupping (0.9 × 0.8), inferior notch, and peripapillary staphyloma; (b) OS with oblique disc, cupping (0.8 × 0.7), and peripapillary atrophy.

Fundus image quality was reduced due to anterior segment changes and the markedly increased axial length of high myopia. In OD, examination revealed a tilted disc with cupping of 0.9 × 0.8, an inferior notch, peripapillary staphyloma, and peripheral laser scars. In OS, the disc was oblique and elongated with cupping of 0.8 × 0.7, 360° peripheral laser marks, and lattice degeneration. Both retinas were attached. Optic disc findings are shown in Figure 2.

**Figure 3:**
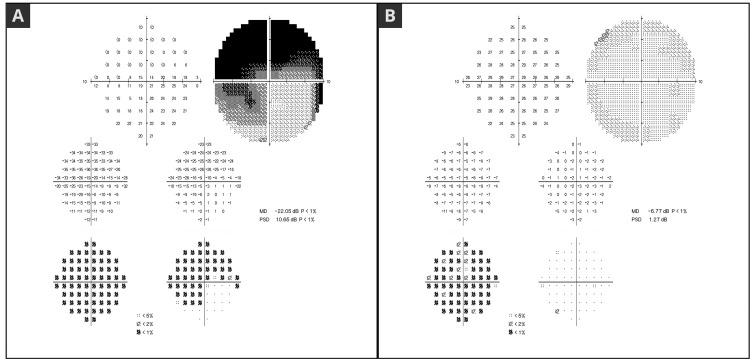
(a) 10-2 visual field of OD showing central island preservation (MD -22.05 dB). (b). 10-2 visual field of OS with mild defects (MD -6.77 dB). Humphrey Field Analyzer (Carl Zeiss Meditec, Dublin, CA) [11]

Due to the severity of the case and the central involvement of glaucomatous damage, we chose to monitor the patient using a central visual field strategy, employing the Humphrey Field Analyzer (Carl Zeiss Meditec, Dublin, CA) 10-2 protocol and SITA-Standard strategy [11]. Figure 3 illustrates a 10-2 visual field report (MD -22.05 dB and PSD 10.65 dB OD; MD -6.77 dB and PSD 1.27 dB OS).

**Figure 4:**
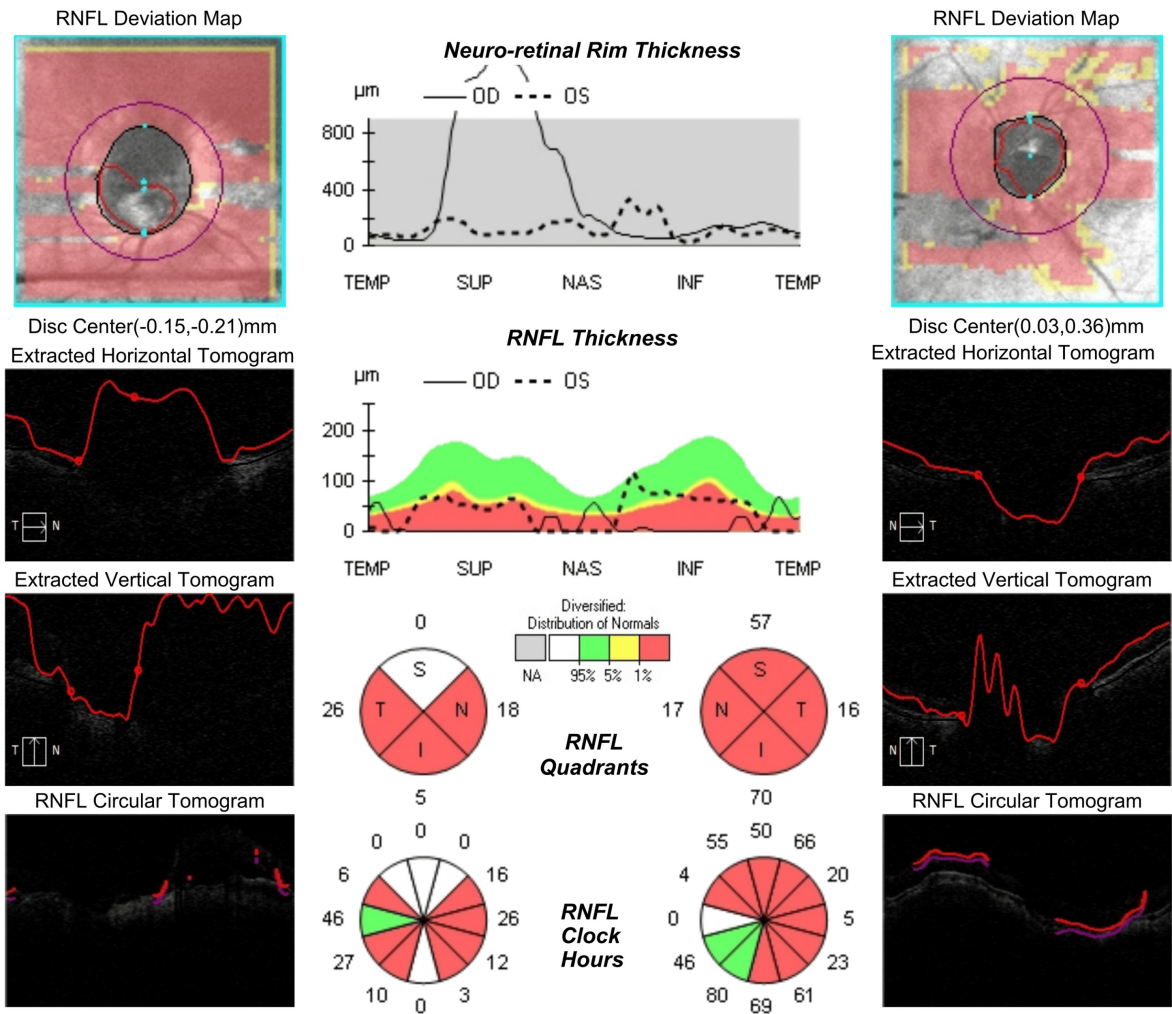
Optical coherence tomography (OCT) of the optic nerve head (ONH) and peripapillary retinal nerve fiber layer (RNFL) in both eyes. RNFL deviation maps, B-scan tomograms (horizontal, vertical, circular), and sectoral RNFL thickness analysis demonstrate diffuse thinning in both eyes. Spectral-domain OCT (Cirrus HD-OCT; Carl Zeiss Meditec) [12]

Structural evaluation by OCT (Cirrus HD-OCT; Carl Zeiss Meditec, Dublin, CA) [12] was markedly limited by the patient’s high axial myopia and advanced glaucoma, which impair segmentation accuracy and distort retinal anatomy. Additional corneal alterations, including prior RK scars and Ferrara intrastromal rings, further degraded optical quality, contributing to irregular astigmatism and anterior segment distortion. Although image acquisition was technically feasible, these factors reduced reliability and explained the variability seen across scans. Consequently, structural metrics were of limited clinical value, and functional testing provided a more dependable measure of progression. Representative retinal nerve fiber layer (RNFL) images from both eyes are shown in Figure 4.

Given concerns about IOP control, Glaub MD® was discontinued at this stage, with continuation of Ganfort® and initiation of Simbrinza® (brinzolamide 1% + brimonidine 0.2%) twice daily.

**Figure 5:**
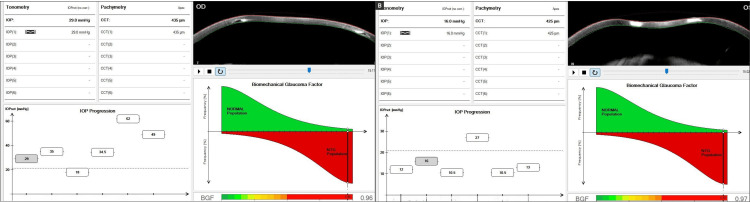
Representation of corneal biomechanical glaucoma diagnostics using the Corvis ST: Vinciguerra Screening Display and Biomechanical Glaucoma Factor (BGF). (a) Corvis ST of the right eye (OD); (b) Corvis ST of the left eye (OS). This image combines two advanced biomechanical assessments derived from Corvis ST (Oculus GmbH) [6]. The Vinciguerra Screening Display provides a multi-parametric analysis including deformation amplitude (DA), stiffness parameter (SP-A1), corneal biomechanical index (CBI), and stress-strain index (SSI), which contribute to risk stratification for ectatic and glaucomatous conditions.

At a subsequent follow-up visit, the patient reported good adherence. Visual acuity remained stable (BCVA 20/40 in OD, 20/30 in OS), but GAT continued to show fluctuations in OD between 14 and 34 mmHg, depending on the applanation site, and 12 mmHg in OS. Corvis ST (Oculus GmbH) [6] demonstrated markedly elevated biomechanically corrected IOP (bIOP) in both eyes (29.1 mmHg OD and 28 mmHg OS), underscoring the limited reliability of GAT in this setting. Oral acetazolamide was initiated at one tablet three times daily. Corneal biomechanical parameters and the biomechanical glaucoma factor (BGF = 0.96) are presented in Figure 5.

Shortly thereafter, the patient showed acute deterioration of the central visual field in the OD (MD -30.07 dB, PSD 6.41 dB), whereas the OS remained stable (MD -7.43 dB, PSD 1.99 dB). Examination revealed an inferior retinal detachment with macula-off configuration. He underwent pars plana vitrectomy with membrane peeling and intraocular gas injection (C3F8). One month after surgery, uncorrected visual acuity in OD was 20/400, with bIOP of 25 mmHg in OD and 12 mmHg in OS. GAT in OD ranged widely from 32 to 50 mmHg, while OS remained near 14 mmHg.

Despite maximal medical therapy, IOP in OD remained uncontrolled. Although applanation readings fluctuated, bIOP stayed consistently high, indicating a persistently elevated biomechanical load. In contrast, OS reached stable pressure control after medication optimization and showed no functional decline, supporting observation rather than intervention. Therefore, micropulse transscleral cyclophotocoagulation (MP-TSCPC, G6 laser) was performed only in the OD. The procedure was carried out over 270° (three quadrants) with 2,500 mW, a 31.3% duty cycle, and continuous sweeping over 360°, sparing the 3 and 9 o’clock meridians, for a total duration of 200-240 seconds. The initial response in OD was modest, with GAT fluctuating between 10 and 28 mmHg; OS remained stable at approximately 15 mmHg. At subsequent visits, OD continued to show wide variation (14-28 mmHg), whereas OS maintained stable pressures around 15-16 mmHg. Months later, OD once again exhibited large fluctuations (24-48 mmHg), while OS remained controlled at 16 mmHg.

A second G6 session was performed with the same laser parameters after documented progression on 10-2 visual field testing, including deepening of defects, reduced foveal sensitivity, decreased VFI, and worsening mean deviation (MD). At the most recent consultation, approximately two weeks after the second G6 laser, BCVA was 20/400 in OD and 20/30 in OS. GAT measured 20 mmHg in OD (notably inferior to the ring segment) and 18 mmHg in OS. Corvis bIOP measured 16.1 mmHg in OD and 15 mmHg in OS. 

The patient remains under close monitoring with maximized medical therapy. Incisional surgery continues to be avoided due to concerns about tissue healing and the limitations it may impose on his competitive athletic activity.

## Discussion

This case illustrates how difficult it can be to diagnose and manage glaucoma in patients with high myopia and a history of refractive surgery. One of the main problems in these situations is obtaining a reliable IOP value. Although GAT is still regarded as the gold standard, it frequently underestimates the pressure in eyes with altered biomechanics, particularly in the presence of RK scars or keratoconus [5,13,14]. In our patient, the cornea in the OD was severely affected by multiple RK incisions and the presence of a Ferrara ring, resulting in significant alterations in corneal structure and biomechanics. In our patient, the cornea had findings usually linked with biomechanical fragility, including reduced pachymetry and a history of RK. However, instead of behaving as expected, its structural response turned out paradoxically rigid. This finding seems to be explained, at least in part, by the presence of the Ferrara ring, which tends to make the stroma locally stiffer. However, it was the sustained intraocular hypertension that most likely played the dominant role in altering the corneal biomechanics, leading to a stiffer, less compliant response on dynamic deformation testing [7]. This suggests that IOP elevation alone can override the expected biomechanical weaknesses conferred by prior surgery and ectasia, especially when chronic or inadequately controlled. In this sense, the Ferrara ring may have contributed to localized corneal resistance. However, it was the high IOP that transformed the entire corneal response into one resembling a structurally robust tissue. GAT readings in OD were highly variable, ranging from 18 to 50 mmHg, depending on the area measured. This variability made it unreliable for clinical decision-making. This case provides an important opportunity to explore the paradox of corneal stiffness observed in structurally compromised eyes. Although the cornea was thin and surgically altered, the biomechanical profile showed unexpectedly high rigidity indices. Corvis ST showed a bIOP of 29.1 mmHg in OD and 28 mmHg in OS. The DA values were 0.69 mm and 0.48 mm, respectively, pointing to reduced deformability. The SSI values were elevated as well, with 3.62 in OD and 3.04 in OS. Taken together, these results suggest that high IOP by itself can alter corneal and overall ocular biomechanics, producing stiffening even in eyes that would usually be considered fragile [14-16]. The DA Ratio (2.35 OD, 1.93 OS) and Corvis Biomechanical Index (CBI: 0.89 OD, 0.00 OS) further emphasized the rigidity profile. Importantly, this rigidity was not predicted by pachymetry alone (475 μm in OD, 482 μm in OS). This case supports the hypothesis that ocular hypertension, even if cases that might be undetected by GAT, may contribute to a secondary biomechanical response of the cornea. A BGF of 0.96 suggests an altered corneal biomechanical pattern, more commonly observed in the glaucomatous population, particularly among patients with normal-tension glaucoma. These results are consistent with the Vinciguerra Screening findings for this patient. As shown in Figure 5, the Corvis ST findings illustrate the bidirectional relationship between IOP and corneal deformation, where sustained ocular hypertension not only results from altered biomechanics but also actively contributes to further biomechanical stiffening, reinforcing the need for tailored diagnostic and therapeutic strategies in such complex cases [7]. In addition, OCT interpretation was hampered by peripapillary atrophy, disc tilt, and segmentation artifacts, which are frequent in high myopia. Consequently, 10-2 visual field testing was adopted as the primary method to monitor progression. Thus, follow-up was guided by bIOP and functional testing, rather than unreliable structural imaging [17].

Corneal biomechanics play a substantial role in tonometry accuracy [5,8]. The Corvis ST, which is a non-contact tonometer that evaluates in vivo the corneal biomechanics profile, uses an air-puff and high-speed Scheimpflug imaging to derive IOP while simultaneously assessing corneal deformation dynamically. Its bIOP algorithm corrects for individual corneal properties [6]. In our case, the bIOP measurement was 29.1 mmHg; however, GAT readings fluctuated markedly, with higher values recorded over the intracorneal ring segment and lower values in uninvolved corneal areas, reflecting heterogeneous corneal biomechanics and rendering GAT clinically unreliable in this post-refractive cornea [3]. Clinicians should be aware that in patients with a history of refractive surgery or corneal ectasia, a “normal” GAT IOP may be falsely reassuring [3].

In glaucoma care, decisions for escalating treatment are often based on a combination of IOP levels, structural damage, and functional loss [1,8]. An important aspect of this case was the need to rely on functional and biomechanical indicators due to the limitations in structural imaging and IOP assessment. In patients with high axial myopia and advanced glaucoma, OCT interpretation is often compromised by anatomical distortions and segmentation errors. In this context, GAT is not expected to underestimate IOP because RK preserves central corneal thickness, unlike refractive procedures that thin the cornea and bias applanation readings downward. Therefore, the marked variability seen here is better explained by the geometric distortion from the intracorneal ring and RK incisions, along with the resulting biomechanical alterations that disrupt the assumptions of applanation tonometry. In this patient, central visual field loss that endangered fixation was documented. Deterioration documented on reliable 10-2 visual field testing provided strong evidence of progression. This finding, together with loss of visual acuity and corroborating biomechanical parameters from the Corvis ST, supported the recommendation for surgical IOP lowering. Patient-reported symptoms, such as decreased contrast sensitivity and impaired color perception during daily activities, reinforced the impression of ongoing visual compromise. In such complex scenarios, conventional parameters may be unreliable. In eyes with prior RK and a Ferrara ring, GAT does not exhibit a predictable bias, as readings can vary due to both corneal irregularity and technique-dependent factors. The integration of objective findings, patient symptoms, and alternative diagnostic strategies ultimately supports a more individualized and rational approach to disease management.

The therapeutic course in this case also provides valuable insights. Traditional incisional glaucoma surgeries in post-RK, high-myopic eyes can be risky. RK incisions can predispose the cornea to instability with any additional surgery; besides, high myopia is associated with thin sclera and increased risk of hypotony maculopathy after filtration surgery [2]. Furthermore, considering the patient’s status as an athlete, there was a strong preference to avoid filtering surgery due to potential postoperative limitations and risks associated with hypotony or physical strain.

We elected to perform MP-TSCPC using the Cyclo G6 laser as an alternative non-invasive treatment. Micropulse cyclophotocoagulation delivers repetitive short bursts of diode laser energy to the ciliary body, reducing aqueous humor production while minimizing collateral tissue damage [18]. Recent studies have shown that MP-TSCPC can safely lower IOP in refractory glaucoma cases, even in eyes with good central vision, with a lower complication rate than traditional continuous-wave cyclophotocoagulation [19]. Varikuti et al. reported a significant IOP reduction (~30% on average) with micropulse TSCPC in a cohort of glaucoma patients, with most maintaining vision and experiencing few adverse effects [18]. In our patient, two sessions of MP-TSCPC were performed over the course of a year, each contributing incremental IOP lowering. With progressive adjustment, the patient’s vision remained stable, without corneal changes, hypotony, or macular edema. Although lower IOP levels would theoretically be desirable in a biomechanically compromised eye, the clinically achieved pressures of approximately 15-16 mmHg were considered acceptable given the measurement bias introduced by altered corneal biomechanics, the balance of treatment risks, and the observed functional stabilization after micropulse therapy. This outcome aligns with the growing consensus that micropulse cyclotherapy is a valuable tool for glaucoma management, especially in eyes where conventional surgery is less feasible [19].

From this case, several clinical pearls can be gleaned. First, in patients with a history of corneal refractive surgery (RK, LASIK, etc.) or ectasia, be cautious in interpreting applanation tonometry, consider supplementary methods like dynamic contour tonometry, or corneal biomechanics assessment (Corvis ST) to ascertain bIOP [20]. Second, monitoring visual field progression remains essential, as functional assessment is an ongoing process that cannot rely exclusively on IOP measurements, particularly when tonometric variability is expected. Third, when intervention is needed, tailor the choice of glaucoma surgery to the individual eye’s condition; less invasive options such as laser cyclophotocoagulation can be advantageous in eyes with compromised ocular integrity. Finally, high-myopic glaucoma patients should be followed long-term with an understanding that their risk for progression remains elevated even at seemingly low IOP, due in part to the vulnerability of the optic nerve and potential measurement errors. A team-based strategy that brings together different specialties can make a real difference. 

In this case, we performed current diagnostic methods with modern treatment strategies. We have achieved a better IOP control with safer outcomes, being primarily relevant in complex patient cases.

## Conclusions

Glaucoma care in post-refractive, highly myopic eyes demands a careful and flexible approach. In this case, GAT did not display the classic underestimation commonly described after RK; rather, it exhibited substantial variability, a consequence of the biomechanical distortion and regional stiffness heterogeneity produced by the refractive surgery and the Ferrara ring segment, making applanation measurements inherently unreliable. This case also illustrates that functional evidence of glaucomatous progression should carry greater clinical weight than uncertain IOP measurements. By combining newer tools, such as bIOP evaluation and micropulse cyclophotocoagulation, we achieved stable pressure control and maintained vision. In short, management of glaucoma in complex eyes with altered corneas must be individualized. Clinicians need to balance innovative ways of measuring pressure with sound therapeutic judgment. Recognizing errors early and adjusting treatment promptly can help prevent irreversible loss of vision. This report also makes clear that, even in advanced and high-risk cases, glaucoma control is still possible when follow-up is close, and the treatment strategy is tailored to the patient.
